# Lipid metabolism dynamics in cancer stem cells: potential targets for cancers

**DOI:** 10.3389/fphar.2024.1367981

**Published:** 2024-06-27

**Authors:** Juan Du, Hai Qin

**Affiliations:** ^1^ Department of Pharmacy, The Affiliated Cancer Hospital of Zhengzhou University and Henan Cancer Hospital, Zhengzhou, China; ^2^ Department of Clinical Laboratory, Beijing Jishuitan Hospital Guizhou Hospital, Guiyang, China

**Keywords:** cancer stem cells, lipid metabolism, fatty acid, lipid droplets, autophagy

## Abstract

Cancer stem cells (CSCs) represent a small subset of heterogeneous cells within tumors that possess the ability to self-renew and initiate tumorigenesis. They serve as potential drivers for tumor initiation, metastasis, recurrence, and drug resistance. Recent research has demonstrated that the stemness preservation of CSCs is heavily reliant on their unique lipid metabolism alterations, enabling them to maintain their own environmental homeostasis through various mechanisms. The primary objectives involve augmenting intracellular fatty acid (FA) content to bolster energy supply, promoting β-oxidation of FA to optimize energy utilization, and elevating the mevalonate (MVA) pathway for efficient cholesterol synthesis. Additionally, lipid droplets (LDs) can serve as alternative energy sources in the presence of glycolysis blockade in CSCs, thereby safeguarding FA from peroxidation. Furthermore, the interplay between autophagy and lipid metabolism facilitates rapid adaptation of CSCs to the harsh microenvironment induced by chemotherapy. In this review, we comprehensively review recent studies pertaining to lipid metabolism in CSCs and provide a concise overview of the indispensable role played by LDs, FA, cholesterol metabolism, and autophagy in maintaining the stemness of CSCs.

## 1 Introduction

**FIGURE 1:**
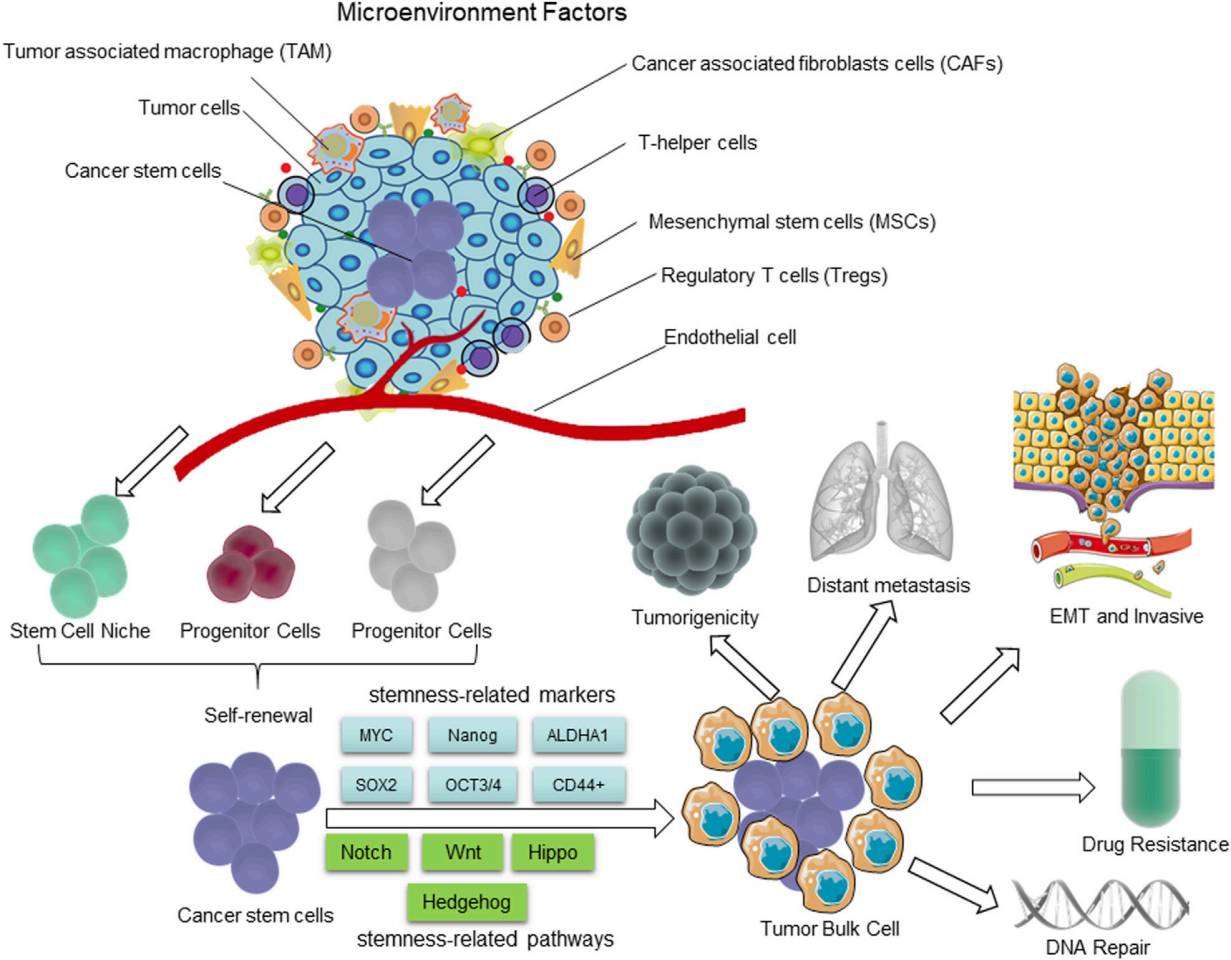
The distinguishing characteristics of CSCs. The presence of CSCs is closely linked to the tumor microenvironment (TME). The self-renewal capability of CSCs is significantly enhanced to certain degrees, comparable to or even surpassing that of normal pluripotent and multipotent stem cells; however, there is a partial loss in their differentiation capacity. During the process of epithelial-mesenchymal transition (EMT), cancer cells acquire characteristics resembling those of stem cells, enabling them to migrate and invade foreign tissues. The hierarchical plasticity subpopulations of CSCs are the primary drivers of tumorigenesis, EMT, tumor progression, metastasis, and drug resistance. The regulation of these CSC characters is mediated by multiple signaling pathways, including Notch, Wnt, Hippo cascades, Hh, *etc.*

The global burden of cancer in 2019 was estimated at 23.6 million new cases (95% UI, 22.2–24.9 million) and 10 million deaths (95% UI, 9.36–10.6 million), making it the leading cause of mortality worldwide (Global Burden of Disease Cancer et al., 2022). Despite advancements in cancer prevention, it remains a formidable challenge for medical professionals due to the scarcity of cures and the prevalence of metastasis or recurrence among patients. The term “stem cells” refers to a unique population of cells that possess the remarkable abilities of self-renewal and differentiation, enabling them to give rise to diverse cell lineages (Al-Hajj and Clarke, 2004). The majority of cancers are composed of heterogeneous cell populations with varying capacities to induce tumor growth. Only the less differentiated and distinct cell populations within the tumor exhibit a high ability to self-renew and initiate tumorigenesis, which are commonly known as cancer stem cells (CSCs), tumor-propagating cells, or tumor initiating cells (TICs), possessing pluripotency and the capacity to repopulate tumors (Reya et al., 2001) (Figure 1). The CSCs represent a limited population of tumor cells exhibiting stem cell characteristics. CSCs serve as the primordial cells responsible for tumorigenesis, recurrence, and metastasis, with numerous CSCs or CSC-like cells having been identified and isolated from diverse tumors (Prasetyanti and Medema, 2017).The identification of CSCs in leukemia was initially achieved through the experimental technique of xenotransplantation (Bonnet and Dick, 1997). The presence of distinct populations of CSCs has been demonstrated in the majority of blood cancers and solid tumors, including breast, brain, colon cancers, and melanoma (Al-Hajj et al., 2003; Singh et al., 2004; Galli et al., 2004; OBrien et al., 2007; Schatton et al., 2008). The presence of CSCs renders them resistant to cytotoxic treatment and significantly contributes to the tumor’s resistance against radio/chemotherapy (Shiozawa et al., 2013). CSCs also initiate metastasis and drive cancer relapse by their capacity for self-renewal and proliferation, enabling them to expand into the bulk of the tumor (Reya et al., 2001). The pressing demand for the advancement of innovative therapies that efficiently eliminate CSCs remains paramount.

The metabolic reprogramming has emerged as a pivotal hallmark of cancer in recent years (Hanahan and Weinberg, 2011). It has been demonstrated that the initiation and progression of cancer are frequently accompanied by profound metabolic alterations (Cao and Yan, 2020; Martinez-Reyes and Chandel, 2021). Notably, the biological characteristics of TICs and CSCs differ from those of non-CSCs (Dando et al., 2015). The metabolic regulation of ATP synthesis and bioconstruct formation in CSCs differs from that of non-stem cancer cells, but resembles that of stem cells derived from normal tissue (Sancho et al., 2016). The role of metabolism in the biology of CSCs has emerged as a prominent area of research over the past decade, with particular emphasis on lipid metabolism. Various studies have demonstrated the crucial role of lipid metabolism in preserving the stemness of CSCs and fulfilling their energy demands, ultimately contributing to cancer progression (Martinez-Outschoorn et al., 2017). For example, the overexpression of acetyl coenzyme a synthase (ACSL1 and ACSL4) and sterol coenzyme a desaturase (SCD) induces epithelial-mesenchymal transition (EMT) in colorectal cancer, thereby enhancing the migratory and invasive capabilities of tumor cells (Sanchez-Martinez et al., 2015). The overexpression of CD36 enhances the uptake of fatty acids and activates Wnt-dependent EMT in hepatocellular carcinoma (HCC) (Nath et al., 2015). Notably, CSCs may originate from normal stem cells or tissue progenitor cells due to stochastic genetic mutations and epigenetic alterations, with tumor progression associated with genome-wide epigenetic regulation influencing CSC maintenance and survival via diverse pathways (Peiris-Pages et al., 2016). The occurrence of abnormal epigenetic modifications can induce the conversion of normal stem cells into CSCs, for example, the processes of DNA methylation and histone modification play crucial roles in guiding the differentiation of stem cells into specific cell and tissue types (Toh et al., 2017). The differentiation of CSCs can be inhibited through the suppression of gene expression via H3K27me3 modification and/or DNA methylation, in a manner analogous to that observed in ESCs (Easwaran et al., 2012). The crucial role of DNA methylation in maintaining the properties of CSCs in leukemia, lung, and colon has been extensively reported (Broske et al., 2009; Brunetti et al., 2017; Maiuri et al., 2019; Liang et al., 2021). Moreover, DNA methylation plays a pivotal role in facilitating this transformation process through the involvement of DNA methyltransferases (Wongtrakoongate, 2015). Notably, these CSC can maintain their stemness through their specific epigenetic alteration by regulating lipid metabolism, for example, CSCs can sustain their stemness through their super-enhancers by promoting polyunsaturated fatty acid (FA) (PUFA) synthesis (Gimple et al., 2019).

Besides, the epigenetic mechanisms also play a crucial role in regulating several key pathways of CSCs, including the Wnt/β-catenin, Hedgehog (Hh), and Notch signaling pathways. These pathways play a pivotal role in the development and maintenance of normal tissues, as well as in the self-renewal and differentiation of hematopoietic stem cells (Hoffmeyer et al., 2012; Myant et al., 2013) (Figure 1). Additionally, they also regulate the proliferation and maintain the stemness of progenitor cells and CSCs in a variety of tissues through modulating lipid metabolic process (Beachy et al., 2004; Yang et al., 2020; Wang et al., 2022). Furthermore, these pathways through which stem cells can be derived via genetic mutations and epigenetic alterations have a significant potential to be exploited for the maintenance of unrestricted proliferation, invasion, and drug resistance (Reya et al., 2001; Wang et al., 2022). This review presents a comprehensive overview of the metabolism of lipid droplets (LDs), FA, and cholesterol, as well as the impact of autophagy on maintaining stemness in CSCs. Furthermore, we investigate the characteristics and mechanisms of lipid metabolism in CSCs and their role in conferring resistance to radiotherapy.

## 2 Biological properties of CSCs

Over the past decade, numerous studies have been conducted to assess the expression profiles of cancer cells exhibiting stem cell properties in various solid tumors, leading to the identification of a plethora of biomarkers, pathways, and therapeutic targets against CSCs (Medema, 2013; Wang et al., 2014). The principal characteristics of CSCs encompass cell surface adhesion molecules, cytoprotective enzymes, transcription factors, and drug efflux pumps (Medema, 2013). However, the markers of CSCs in one organ or tissue differ from those in other organs or tissues, with only a few shared markers between them. Table 1 provides an overview of the most prevalent molecules that can serve as CSC biomarkers. Recently, the involvement of CSCs is pivotal in driving cancer progression, facilitating metastasis, promoting recurrence, and conferring resistance to cytotoxic therapies. The primary targets of classical radiotherapy and chemotherapy are predominantly fast-proliferating cells (Gerlinger et al., 2012). Unlike normal stem cells, cancer stem cells are believed to be responsible for tumor growth, recurrence, and drug resistance. One of the key characteristics of stem cells is their quiescent or dormant state, indicating infrequent division and prolonged periods in a dormant state. This attribute renders them less susceptible to conventional cancer therapies that primarily target rapidly dividing cells. However, recent studies have indicated that certain CSCs expressing leucine-rich repeat G protein-coupled receptor 5 (LGR5+) may not exhibit complete quiescence (Shiokawa et al., 2020). LGR5+ CSCs have been identified in various types of cancers, including colorectal, liver, and pancreatic cancers. It is believed that these cells undergo regular cell division cycles, indicating their cyclic nature. These findings hold significant implications as they challenge the previous notion of cancer stem cells being quiescent and suggest that targeting these cells may be more effective than conventional therapies aimed at rapidly dividing cells (Higa and Nakayama, 2024). Furthermore, it underscores the importance of comprehending the heterogeneity within the CSC population since different subpopulations may exhibit varying degrees of quiescence and sensitivity to treatment. In conclusion, while the concept of CSCs existing in a dormant state has long been fundamental in cancer research, recent evidence suggests that at least some CSCs, such as LGR5+ CSCs, are not entirely dormant. This discovery carries crucial implications for developing novel therapeutic strategies to target these cells and enhance cancer prognosis.

**TABLE 1:** The summary of diverse markers utilized for the identification of CSCs.

Tumor type	CSCs markers	Biological function	Reference
hepatocellular carcinoma, lung, melanoma, pancreatic	ABCG2	The ABCG2 protein is an ATP-binding cassette transporter primarily involved in drug metabolism and cellular drug resistance, potentially contributing to the development of chemotherapeutic drug resistance in tumor cells	Herpel et al. (2011), Jia et al. (2013), Su et al. (2016)
Breast, Colorectal, Esophageal, Glioblastoma, Liver/Lung adenocarcinoma, Nasopharyngeal	SOX2	The dysregulated expression of SOX2 has been implicated in various cancer types, and research studies have demonstrated that SOX2 exerts a positive influence on key characteristics of cancer cells, including proliferation, migration, invasion, and metastasis. In addition, SOX2 mediates resistance to existing cancer therapies and is expressed in CSCs	Novak et al. (2020)
leukemia, liver, colorectal, prostate, ovarian, lung, head and neck, brain, pancreatic, gastric and breast cancer	Nanog	The transcription factor Nanog is widely recognized as a pivotal marker for the identification of CSCs. The activation of Nanog via distinct signaling pathways, such as JAK/STAT and Wnt/β-catenin cascades, elicits stemness, self-renewal capacity, metastatic potential, invasiveness, and chemoresistance in cancer cells	Vasefifar et al. (2022)
Colorectal, head and neck, Lung, pancreatic cancer	CD166 (ALCAM)	CD166 (ALCAM) is a cell surface molecule that is a member of the immunoglobulin superfamily. CD166 is widely expressed in various tumors, and its biological functions in tumor stem cells primarily encompass the facilitation of tumor growth and metastasis, enhancement of tumor cell survival, modulation of immune response, involvement in the formation of the tumor microenvironment, as well as regulation of self-renewal and differentiation processes within tumor stem cells	Jiao et al. (2012), Yan et al. (2013a), Margaritescu et al. (2014)
Breast, lung, colorectal, liver, gastric, cervical, esophageal, ovarian, head, and neck cancer	ALDH1	The expression of ALDH1 is considered as a reliable indicator for the presence of cancer stem cells (CSCs), which play a crucial role in tumorigenesis by preserving CSC properties, modulating cellular metabolism, and facilitating DNA repair mechanisms	Wei et al. (2022)
Breast, colorectal, glioma, liver, lung, ovarian, pancreatic, prostate	CD44	The cell surface glycoprotein functions as a receptor for various extracellular matrix components, such as acid hyaluronic, collagen, integrins, and metalloproteinases, thereby facilitating cellular migration and self-renewal	Todaro et al. (2014), Yan et al. (2015), Erhart et al. (2019), Herreros-Pomares et al. (2019)
Breast, glioma, liver, lung	CD90 (THY1)	The involvement of a highly conserved glycophosphatidylinositol (GPI)-anchored cell-surface eggplant protein in T cell adhesion and signaling, promotion of tumor growth and metastasis, as well as regulation of self-renewal and differentiation of tumor stem cells has been observed	Yan et al. (2013b)
Colorectal, liver, lung, ovarian, prostate	CD326	The transmembrane glycoprotein CD326, also known as EpCAM (Epithelial Cell Adhesion Molecule), is predominantly expressed on normal epithelial cells and functions as a homotypic calcium-independent cell adhesion molecule. It plays crucial roles in tumor stem cells by promoting cell proliferation, inhibiting apoptosis (programmed cell death), and maintaining stem cell properties. This makes CD326 an important therapeutic target.	Sayd et al. (2014)
Colorectal, Breast, lung, prostate	Integrinα6β4	A cell adhesion molecule that specifically binds to laminin in the extracellular matrix and initiates the formation of semimembranous vesicles, thereby facilitating cellular migration and invasion, has been found to be closely associated with the proliferation, invasion, and metastasis of tumor stem cells. Integrin α6β4 interacts with the extracellular matrix, promoting survival and proliferation of tumor stem cells while aiding in evading immune system surveillance by tumor cells	Subramaniam et al. (2018)

**FIGURE 2:**
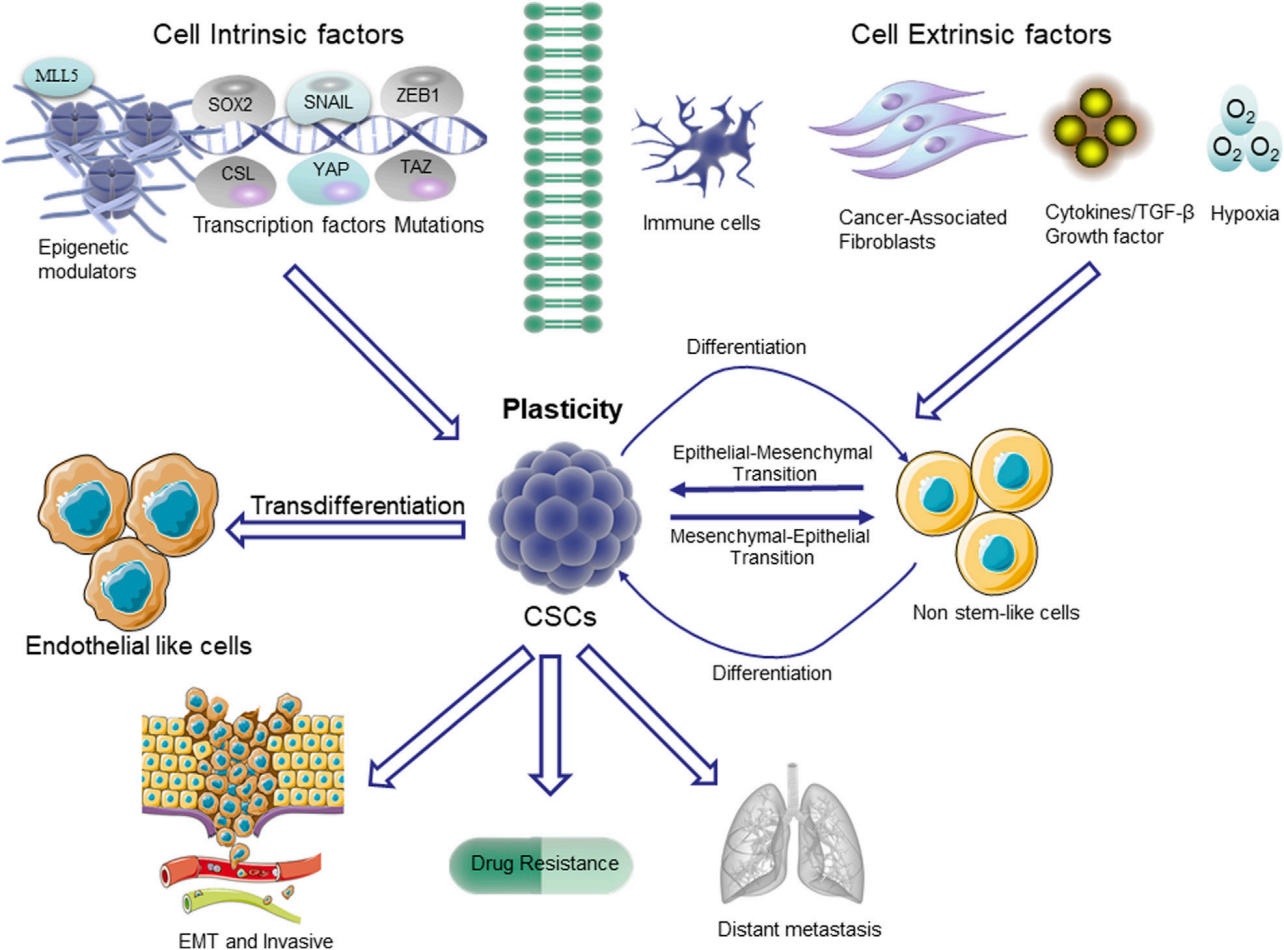
Plasticity in CSCs. The non-stem cells depicted in the figure typically exhibit restricted tumorigenic capacity and maintain their differentiated state. The upregulation of the stem cell pathway in these cells enables them to undergo a transition to a higher level of cellular state, referred to as plasticity. The pathways that trigger stem cell differentiation are mediated through transcription factors involved in epithelial-mesenchymal transition (EMT), such as SNAIL and ZEB1, as well as stem cell-specific transcription factors like SOX2. Additionally, the Hippo/YES-associated protein (YAP)/tafazzin (TAZ) signaling pathway, NOTCH/CSL pathway, and the epigenetic regulator MLL5 also play crucial roles in this process.

It is widely recognized that one of the primary challenges in cancer treatment lies in the development of drug resistance. Cellular plasticity has been identified as a pivotal factor contributing to the emergence of drug resistance (Boumahdi and de Sauvage, 2020). Cancer cell plasticity refers to a state wherein non-transformed differentiated cells exhibit adaptive plastic behavior under oncogenic stress (Figure 2). This phenomenon promotes diversity and heterogeneity among cancer cells within tumors, serving as a bypass mechanism to evade therapeutic agents. In the context of Darwinian selection, a vast number of non-CSCs conceals a small population of CSCs. According to the coexisting model theory, dynamic transcriptional fluctuations in individual cells give rise to therapeutic resistance markers, leading to resistance against treatment. The Lamarckian induction concept suggests that epigenetic modifications occurring in a small subset of cancer cells result in alterations of their drug-refractory phenotype and subsequently enhance therapeutic resistance. These changes in phenotype are commonly referred to as cellular plasticity (Vergara et al., 2019; Paul et al., 2022). This phenomenon promotes diversity and heterogeneity of CSCs within the tumor as a bypass mechanism to evade therapeutic agents.

In addition, CSCs express high levels of ATP-binding cassette transporters (ABC transporters), which contribute to the efflux of chemotherapeutic agents, leading to multidrug resistance (Dean et al., 2005; Robey et al., 2018). In order to develop novel and efficacious therapeutic strategies targeting the stem cell-like subpopulations of tumor cells, it is imperative to gain comprehensive insights into the characteristics of CSCs and elucidate the underlying mechanisms responsible for their acquired resistance and stem cell-like properties, which are closely associated with CSCs plasticity, senescence, and quiescence. The concept of plasticity refers to the phenomenon that stem cells can generate tumor cells through asymmetric division (Vlashi and Pajonk, 2015). However, this hierarchy is not unidirectional; tumor cells have the ability to undergo de-differentiation and acquire stem-like properties (Figure 2). The occurrence of plasticity can be attributed to genetic and epigenetic alterations. For example, the non-CSC tumor cells in the basal-like subtype of breast cancer have the ability to undergo a transition to a CSC-like state through ZEB1 (Chaffer et al., 2013). In glioblastoma (GBM), cancer cells expressing CSC markers do not represent a functionally distinct clonal entity but rather exhibit a phenotypic plasticity that can be induced by microenvironmental cues (Dirkse et al., 2019). Recent findings from single-cell RNA sequencing analyses of human GBM tumors have revealed intratumoral heterogeneity, indicating the simultaneous presence of three cancer grades and cellular plasticity within the tumors (Patel et al., 2014; Neftel et al., 2019; Couturier et al., 2020; Lopes and Vinga, 2020). The study conducted by Neftel reveals that plasticity encompasses not only the process of de-cellularization but also the dynamic transition between distinct cell states, including intratumoral heterogeneity. This is exemplified by the simultaneous presence of 4 cell states within a single GBM, namely, neural progenitor cells, oligodendrocytes, astrocytes, and mesenchymal-like cells, which can interconvert among each other (Neftel et al., 2019). The rapid changes in cellular state can significantly impede therapies aimed at targeting specific tumor cell states. Cellular senescence is a stress response triggered by various molecular damages, resulting in cell cycle arrest, and characterized by diverse phenotypic alterations, including the secretion of bioactive molecules. Senescent cells progressively accumulate during the aging process and are observed in cancerous and fibrotic lesions (Bousset and Gil, 2022). The process of cellular senescence exerts a well-established tumor-suppressive effect by constraining the tumorigenic potential of cancer cells and enhancing the efficacy of cytotoxic therapies (Braig et al., 2005; Michaloglou et al., 2005; Dorr et al., 2013). However, the co-regulation of senescence and stemness functions through overlapping signaling pathways such as p16, p21, and p53 suggests that senescence may trigger genetic reprogramming and activate stemness, thereby contributing to CSC-mediated tumor progression, metastasis, and therapy resistance (Zon, 2008; Milanovic et al., 2018). A recent study has also provided insights into dysfunctional aging from a molecular perspective: in response to genotoxic damage or oncogenic stresses, tumor cells activate extensive chromatin remodeling that involves the addition of repressive methylation marks. The aforementioned marks exert a stable repression on S-phase promoter genes, while concurrently enhancing the secretion of pro-tumorigenic factors and activating stem cell transcription factors such as WNT/lymphocyte enhancer factor 1 (LEF1) (Lecot et al., 2016; Milanovic et al., 2018). The elucidation of the pathways involved in this reprogramming mechanism during cellular senescence will facilitate the overcoming of challenges posed by current therapeutic approaches through targeted CSCs.

The ability of CSCs to transition into a dormant or quiescent state, specifically entering the reversible G0 phase of the cell cycle and remaining dormant, is another distinguishing characteristic (Chen W. et al., 2016). This state transition is typically induced by the microenvironment, such as hypoxia, nutrient deprivation, or oxidative stress, or by selective pressure from chemotherapeutic agents. The fact that most conventional cancer treatments primarily target actively dividing cells allows quiescent CSCs to evade the effects of such therapies and tend to transition back into a proliferative state when favorable conditions arise (Sosa et al., 2014; De Angelis et al., 2019). The resistance of slow-circulating CSCs to temozolomide treatment in GBM was determined through strain tracing. Interestingly, the restoration of sensitivity to temozolomide was observed upon ablation of this specific population (Chen et al., 2012). The future plays a crucial role in the successful treatment of cancer through the identification of CSCs derived from quiescent or dormant cell populations and their associated maintenance pathways.

## 3 Metabolic properties of CSCs

Due to the heterogeneity of tumor cells, cancer cells heavily rely on glucose and aerobic oxidation for energy supply, distinguishing their energy metabolism from that of normal cells (Liberti and Locasale, 2016). The reprogramming of metabolism is a hallmark feature exhibited by cancer cells (Hanahan and Weinberg, 2011). The metabolic characteristics of cancer cells differ significantly from those of normal cells. Given the hypoxic, highly oxidized, acidic, and nutrient-deprived TME resulting from rapid tumor cell proliferation and inadequate blood vessel formation, cancer cells must effectively adapt their cellular bioenergetics to survive in this unfavorable milieu (Viale et al., 2014; Lue et al., 2017). This adaptive process is commonly referred to as metabolic reprogramming. Metabolic reprogramming becomes indispensable for sustaining cancer cell proliferation and survival when oncogenic signaling is obstructed. The majority of human cancers exhibit aerobic glycolysis even in the presence of abundant oxygen, a phenomenon commonly referred to as the Warburg effect (Danhier et al., 2017; Strickland and Stoll, 2017). The Warburg effect characterizes the metabolic shift from oxidative phosphorylation (OXPHOS) to glycolysis in the pentose phosphate pathway, accompanied by the substitution of lactate accumulation for sustained ATP production in the TME (Koren and Fuchs, 2016). The metabolic rewiring not only fulfills the energy demands for sustained proliferation, but also generates a substantial amount of substrate for cellular compartments. It has been shown that poorly differentiated cancers exhibit higher glucose uptake than differentiated cancers, suggesting that the high glycolytic flux in tumor tissues arises mainly from impaired differentiation of CSCs (Riester et al., 2018). The activation of mitochondrial metabolism, in contrast, results in the loss of pluripotent potential and triggers the differentiation of P19 embryonal carcinoma stem cells (Vega-Naredo et al., 2014). The emerging evidence suggests that the Warburg effect of glycolytic metabolism is implicated in the processes of stemness and EMT (Aguilar et al., 2016). Further findings also provided compelling evidence that the regulation of stem cell metabolism plays a pivotal role in governing the control of stem cell fate. For instance, the compound R406 functions as a Syk inhibitor in immune thrombocytopenia by inducing a metabolic shift from glycolysis to OXPHOS in glioma stem cells (GSCs). This metabolic alteration subsequently leads to an excessive production of reactive oxygen species (ROS), ultimately triggering apoptosis in GBM cells (Sun et al., 2019). Peng et al. demonstrated that the overexpression of pyruvate dehydrogenase kinase 1 (PDK1) in breast CSCs (BCSCs) leads to the inhibition of aerobic glycolysis in mitochondria. The depletion of PDK1 resulted in a significant reduction in ALDH1-positive BCSCs, thereby impairing their ability to form spheroids (Peng et al., 2018). During the process of EMT in basal-like breast cancer, SNAIL-mediated methylation of the fructose-1,6-bisphosphatase promoter enhances the characteristics of CSCs and tumorigenicity by increasing glucose uptake and macromolecule biosynthesis. Additionally, it inhibits oxygen consumption through the inhibition of mitochondrial complex I activity (Dong et al., 2013). The presence of mutations in the internal tandem duplication (ITD) of the Fms-like tyrosine kinase 3 gene (FLT3/ITD) in acute myeloid leukemia (AML) is considered an unfavorable genetic alteration associated with a poor prognosis (Burchert, 2021). H-Q Ju et al. reported that FLT3/ITD induces a significant increase in aerobic glycolysis through akt-mediated upregulation of mitochondrial hexokinase 2, leading to heightened reliance on glycolysis and enhanced sensitivity of leukemic cells to pharmacological inhibition of glycolytic activity. The preferential inhibition of glycolysis results in severe depletion of ATP and extensive cell death in FLT3/ITD leukemia cells (Ju et al., 2017).

Notably, although the utilization of glycolysis for survival has been reported in some studies, others have suggested that CSCs may also rely on OXPHOS for their survival (Ciavardelli et al., 2014; Pasto et al., 2014). The transfer of electrons and H+ from the donor molecules, reduced nicotinamide adenine dinucleotide or reduced flavin adenine dinucleotide, to the acceptor molecule O_2_ is accompanied by the liberation of energy for ATP synthesis, involving a series of protein complexes located in the inner mitochondrial membrane. LAGADINOU et al. discovered that AML cells enriched with low levels of ROS from leukemia stem cells (LSCs) exhibited elevated expression of B-cell lymphocytoma-2 (Bcl-2), an antiapoptotic protein involved in mitochondrial regulation. Furthermore, Bcl-2 inhibition hampers ATP production in leukemia stem cells (LSCs) by hindering oxidative phosphorylation. Unlike AML cells and normal CD34^+^ cells, LSCs lack efficient backup glycolysis, making this metabolic vulnerability a promising target for selective elimination in clinical applications (Lagadinou et al., 2013). Additionally, through isotope tracing combined with metabolomics, researchers have demonstrated that LSCs exhibit enhanced efficiency in converting stearic acid and glucose into intermediates of the tricarboxylic acid cycle compared to other chronic myeloid leukemia cells, indicating a high dependence on OXPHOS (Kuntz et al., 2017). Similarly, the crucial significance of OXPHOS in solid tumors persists for CSCs. Ciavardelli et al., 2014 demonstrated that the proliferation of CD44+/CD24-breast CSCs could be reduced by inhibiting their glycolysis, indicating a glycolytic nature of this specific population. The proto-oncogene-encoded transcription factor MYC and the anti-apoptotic protein MCL1 synergistically enhance OXPHOS in CSCs, thereby promoting chemoresistance maintenance in triple-negative breast cancer (TNBC) (Lee et al., 2017). The results of clinical studies also indicate that well-differentiated tumors exhibit decreased uptake levels of 18F-fluorodeoxyglucose, whereas poorly differentiated tumors demonstrate elevated uptake levels of 18F-fluorodeoxyglucose (Riester et al., 2018). Additionally, the levels of oxidative metabolism and ATP in GSCs are higher compared to differentiated tumor cells and inhibiting OXPHOS, but not glycolysis, significantly impairs the tumorigenic potential and survival ability of GSCs in xenograft models (Minami et al., 2021). Notably, the nutrient-deprived CSCs in GBMs preferentially utilize the pentose phosphate shunt, thereby facilitating the self-renewal, proliferation, and survival of CSCs (Kathagen et al., 2013). Furthermore, the regulation of OXPHOS in GSCs is mediated by endogenous insulin-like growth factor 2 mRNA binding protein 2 (IMP2), which plays a crucial role in the transportation of mRNAs encoding respiratory chain-associated components to the mitochondria and facilitates OXPHOS maintenance through its involvement in the assembly of respiratory chain complexes (Janiszewska et al., 2012). In another study, based on the energy metabolism characteristics of pancreatic CSCs, a shift in the carbon source was employed by replacing glucose with galactose to induce enhanced OXPHOS activity in pancreatic cancer cells under *ex vivo* conditions. Consequently, this approach led to the enrichment of pancreatic CSCs, which exhibit upregulated expression of CSC surface antigens, heightened tumorigenicity, and immune evasion properties (Valle et al., 2020). Importantly, nasopharyngeal carcinoma, ovarian cancer, osteosarcoma, GBM, and colon cancer heavily rely on mitochondrial OXPHOS for energy generation (Zhou et al., 2011; Liao et al., 2014; Palorini et al., 2014; Shen et al., 2015; Song et al., 2015). These findings suggest that targeting aerobic glycolysis or OXPHOS could be a potential strategy for eradicating CSCs.

## 4 Key modulators of lipid metabolism in CSCs

**FIGURE 3:**
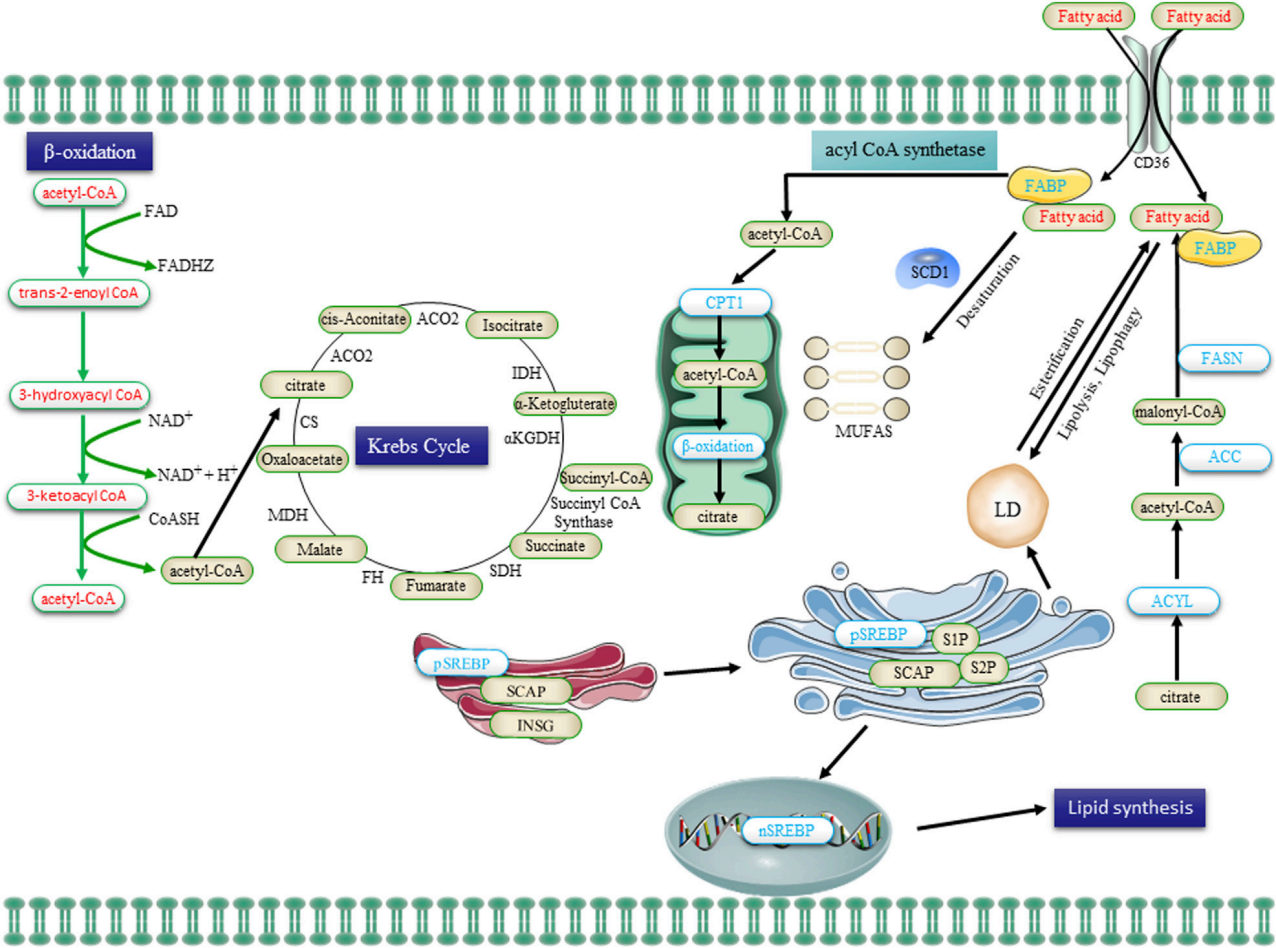
The lipid metabolism in CSCs, encompassing uptake, lipogenesis, and utilization, undergoes alterations. Extracellular FA is transported into cells via the CD36 receptor and subsequently undergone β-oxidation in mitochondria to generate acetyl-CoA. Acetyl-CoA is then converted to citrate by the enzyme citrate synthase, which enters the Krebs cycle for complete oxidation. The *de novo* synthesis of FA initiates with acetyl-CoA and proceeds through the sequential addition of two-carbon units, a process tightly regulated by the key enzyme FASN. Transcriptional control exerted by SREBPs governs the majority of enzymes involved in FA synthesis. Saturated FA undergoes desaturation to form monounsaturated FA (MUFA) catalyzed by SCD1. Additionally, FA is esterified to glycerol and subsequently stored as triglycerides in LDs. FA, obtained from the uptake of exogenous lipids or through *de novo* lipogenesis, is released to provide cellular fuel via FAO in the mitochondria, with CPTI catalyzing the rate-limiting step. Abbreviations: FA, fatty acid; FABP, FA binding protein; FASN, FA synthase; ACC, acetyl-CoA carboxylase; SREBP, sterol regulatory element binding protein; SCAP, SREBP cleavage-activating protein; INSG, insulin-induced gene protein; SCD1, stearoyl-CoA desaturase 1; MUFAs, mono-unsaturated FA; CPT1, carnitine palmitoyltransferase 1; and LDs, lipid droplets.

The alteration of cellular metabolism, particularly in lipid metabolism, has recently been acknowledged as a distinctive characteristic of various cancer cells (Yi et al., 2018). The lipid category encompasses various types of lipids, such as phospholipids, cholesterol and cholesterol esters, while fats primarily refer to triglycerides (TG). Lipids play a crucial role in numerous cellular functions, including membrane formation, signaling pathways, and other biological activities, while TG serves as a significant source of cellular energy (Lingwood and Simons, 2010). A growing body of evidence suggests that cancer cells undergo alterations in various aspects of cell membrane formation, lipid synthesis and degradation, as well as lipid-driven cellular signaling. A hallmark of cancer metabolism is the upregulation of *de novo* lipogenesis (Kinlaw et al., 2016), as illustrated in (Figure 3). The energy requirements of cancer cells are primarily met through *de novo* lipogenesis, as dietary lipids are limited in availability, unlike most non-malignant cells. The metabolic intermediates of glycolysis may be redirected towards enhanced lipid biosynthesis by CSCs, thereby promoting self-renewal growth (Corominas-Faja et al., 2014). The expression of various lipid synthases is elevated in cancer cells, including sterol regulatory element binding proteins (SREBPs), ATP-citrate lyase (ACLY), acetyl-CoA carboxylase (ACC), fatty acid synthase (FASN), and stearoyl-CoA desaturase 1 (SCD1) (Currie et al., 2013; Sun et al., 2016; Geng et al., 2016; Rohrig and Schulze, 2016; Shimano and Sato, 2017). The citric acid (TCA) cycle also plays a crucial role in lipid metabolism by providing acetyl groups for FA synthesis, thereby contributing to the maintenance of malignant biological behavior in cancer cells (Williams and ONeill, 2018). Here, we will concentrate the roles of these master regulatory elements in CSC progression.

### 4.1 ACC

The activation of ACC leads to the catalysis of the conversion from acetyl coenzyme A (AcCoA) to malonyl-coenzyme A, which plays a crucial role in *ab initio* FA synthesis, particularly when cells require additional FA for energy demands or other biosynthetic processes. CSCs typically exhibit a heightened capacity for FA synthesis owing to their substantial energy demands required to sustain their highly active metabolic state (Liu et al., 2022). Additionally, ACC exhibits high expression levels in induced pluripotent stem cells (iPSCs) and suppression of ACC expression significantly diminishes the reprogramming efficiency of iPSCs (Vazquez-Martin et al., 2013). As normal stem cells always share the similar reprogramming procedure with that of CSCs (Zheng et al., 2019), this pronounced upregulation of ACC and FASN in iPSC underscores the significance of adipogenesis in stem cells and paves the way for potential therapeutic applications for CSCs. Thus, targeting ACC has been shown to suppress CSC progression, such as the ACC inhibitors *in vitro* reinstated the tumor cells to a histological epithelial phenotype (Petrova et al., 2017), and the inhibition of ACC in pancreatic cancer cells effectively suppresses both *in vivo* and *in vitro* pancreatic tumor growth by attenuating the ligand palmitoylation of Wnt and Hh, thereby inhibiting the signaling pathways of Wnt and Hh (Petrova et al., 2017), both of which are also implicated in the regulation of ACC in CSCs, as evident by that β-catenin knockdown leads to an upregulation of ACC expression (Vergara et al., 2017). Additionally, the inhibition of ACC activation effectively restored intracellular lipid levels, attenuated EMT, and suppressed the characterization of CSCs (Bort et al., 2020). Currently, given its pivotal role in lipid metabolism within CSCs, ACC represents a promising target for potential therapeutic interventions against numerous tumors (Moncur et al., 1998; Fang et al., 2014; Svensson et al., 2016).

### 4.2 ACLY

Located predominantly in the cytoplasm, ACLY facilitates the enzymatic conversion of citric acid into AcCoA and oxaloacetate, thereby playing a crucial role in FA and cholesterol synthesis within CSCs (Feng et al., 2020). AcCoA serves as a crucial substrate not only for FA and cholesterol synthesis, but also plays an essential role in protein acetylation reactions, consequently emerging as a pivotal enzyme in lipid synthesis and connecting catabolic pathways with biosynthesis (Metallo et al., 2011). The upregulation and activation of ACLY have been extensively documented in various malignancies, as evidenced by the association with an increased malignant phenotype and a poor prognosis (Hatzivassiliou et al., 2005; Qian et al., 2015). Additionally, ACLY can facilitate tumor stemness through the downstream effectors, such as overexpression of ACLY enhances the expression of Snail, an EMT master regulator, thereby promoting EMT and stemness (Hanai et al., 2013). Conversely, inhibition of ACLY diminishes the invasiveness of breast cancer cells, while targeting ACLY reduces the proliferative potential and cisplatin resistance of ovarian cancer cells (Lucenay et al., 2016; Wei et al., 2021). Notably, Migita T et al. discovered a positive correlation between phosphorylation of ACLY in the PI3K/AKT pathway and the stage, grade of tumor differentiation, as well as poor prognosis in non-small cell lung cancer. This particular form of phosphorylation is believed to significantly enhance the role of ACLY in CSCs (Migita et al., 2008).

### 4.3 CD36

The transmembrane glycolipid protein CD36 is extensively expressed in various cell types, encompassing adipocytes, myocytes, endothelial cells, macrophages, and hepatocytes (Yang P. et al., 2022). CD36 protein exhibits numerous biological functions, encompassing lipid metabolism, inflammatory response, apoptosis, cell migration, and tumorigenesis (Zhao L. et al., 2018; Wang and Li, 2019). Elevated CD36 expression in tumor cells plays a pivotal role in lipid metabolism by facilitating the uptake and utilization of saturated fatty acids, such as palmitic acid, thereby contributing to both lipid synthesis and catabolism processes and augmenting proliferation, invasion, and metastatic potential (Li et al., 2022; Yuan et al., 2022). Additionally, CD36 is implicated in the crucial FA metabolic pathway known as β-oxidation (Zeng et al., 2022). Consistently, the blockade of β-oxidation through the targeting of CD36 with neutralizing antibodies has the potential to completely eradicate metastasis in melanoma and breast cancer (Pascual et al., 2017). In GSCs, CD36 facilitates the uptake of oxidized phospholipids, thereby promoting the proliferation of GSCs (Hale et al., 2014). Specifically, CD36 recognizes and binds to oxidized phospholipids, internalizing them into the intracellular compartment, thereby facilitating the proliferation and self-renewal of GSCs, this is attributed to the inhibitory effect exerted by elevated CD36 levels on the activation of apoptotic signaling pathways, thereby safeguarding GSCs against death-inducing stimuli. Additionally, with respect to non-solid tumor hematopoietic stem cells, CD36-enriched LSCs derived from gonadal adipose tissue exhibit enhanced survival advantages and resistance to treatment (Ye et al., 2016).

### 4.4 FASN and FA binding protein (FABP)

Recently, it has been demonstrated that lipids and lipoproteins, both acquired through exogenous (or dietary) uptake and synthesized endogenously, exert a profound influence on the maintenance of CSCs’ stemness during tumorigenesis. FASN, a key enzyme in *de novo* lipid synthesis, has consistently been identified as a facilitator in various types of CSCs (Ali et al., 2018; Rabionet et al., 2021; Castagnoli et al., 2023). Interestingly, overexpression of FASN in patient-derived GSCs was significantly diminished during serum-induced differentiation, indicating that augmented *de novo* adipogenesis contributes to the maintenance of the undifferentiated state of GSCs. After treatment with 20 μM cerulenin, a pharmacological inhibitor of FASN, the proliferation and migration of GSCs were significantly suppressed, and *de novo* lipogenesis was reduced. Additionally, the expression levels of nestin, Sox2, and FABP7, which are markers of GCSs, are decreased while the expression level of glial fibrillary acidic protein increased (Yasumoto et al., 2016). However, to date, limited research has been conducted regarding the involvement of FABPs in CSCs. The FABPs, functioning as lipid chaperones, are believed to bind and transport FA across various cellular compartments and organelles including plasma membranes, mitochondria, LDs, endoplasmic reticulum, and nuclei. A notable exception is FABP7, a widely recognized neural stem cell marker that exhibits high enrichment in GSCs and demonstrates significant downregulation in differentiated GSCs (Morihiro et al., 2013). Furthermore, Antonella De Rosa et al. showed that FABP7 downregulation in GSCs is associated with decreased proliferation and migration ability (De Rosa et al., 2012). Through proteomic and metabolomic analyses, Brandi et al. demonstrated that pancreatic CSCs exhibit elevated glycolysis levels and increased *de novo* adipogenic activity, while displaying reduced mitochondrial OXPHOS levels compared to a significant number of parental cancer cells. The authors discovered that FASN exhibited higher expression levels in Panc1 CSCs and displayed increased sensitivity to cerulenin inhibition compared to parental non-stem cell cancer cells (Brandi et al., 2017). Additionally, the expression level of FASN is regulated by β-catenin and exhibits a positive correlation with the expression levels of stem cell markers (SOX2, CD133, and Nestin) in GSCs (Yasumoto et al., 2016; El-Sahli et al., 2019). Importantly, FASN inhibitors have been shown to decrease the expression of stemness markers in GSCs (Yasumoto et al., 2016). For instance, resveratrol has the ability to induce apoptosis in CSCs by inhibiting adipogenesis through the downregulation of FASN expression (Pandey et al., 2011). Notably, CSCs often show a positive correlation between the levels of FASN and ACC expression under specific conditions, and FASN is more vulnerable to attacks in CSCs compared to regular cancer cells. Ongoing molecular and cell-based preclinical studies have focused on the development and characterization of various FASN blockers. Despite these efforts, translating these promising findings into clinical applications remains a challenging endeavor (Menendez and Lupu, 2017; Schcolnik-Cabrera et al., 2018). Therefore, numerous efforts should be directed towards several key areas. Firstly, there is a need for a comprehensive investigation into the interplay between FASN and ACC expression in CSCs to enhance our understanding of their correlation in CSCs. Additionally, a deeper exploration of the mechanisms underlying the increased vulnerability of FASN in CSCs compared to normal cancer cells is essential for the development of more effective therapeutic strategies. Optimization of existing FASN blockers is crucial, ensuring they exhibit high selectivity and efficacy in inhibiting FASN within CSCs. Moreover, emphasis should be placed on translating molecular and cell-based preclinical research findings into clinical applications by designing improved drug delivery systems and refining precision treatment protocols. Finally, addressing challenges associated with clinical applications, such as formulating suitable treatment regimens, ensuring drug safety and efficacy, and accommodating individual patient variations, is vital for the successful implementation of these research outcomes in the clinical setting. Through these concerted efforts, a better understanding of FASN’s role in CSCs can be achieved, leading to improved treatment strategies and ultimately facilitating the translation of these research findings into clinical applications.

### 4.5 Lipid Stearoyl-CoA desaturase-SCD1

Recently, multiple studies have demonstrated that an elevation in FA within cancer cells may also heighten their reliance on desaturase activity (Peck and Schulze, 2016). The promotion of CSCs through the regulation of unsaturated FA has been demonstrated in several studies, highlighting the significance of signaling pathways in this process, for example, NF-κB, a pivotal regulator of tumors and CSCs, directly governs the expression and activation of lipid desaturases (Li et al., 2017). Moreover, inhibition of adipogenesis through desaturase inactivation effectively disrupts the AKT/ERK-mediated NF-κB signaling pathway (Fritz et al., 2010; Li et al., 2017). Similarly, the levels of SCD-dependent MUFAs directly regulate CSCs through the Wnt/β-catenin pathway, which is a pivotal signaling pathway in stem cells and CSCs (Zheng et al., 2016; Lai et al., 2017). The two isoforms of SCDs in humans are SCD1 and SCD5, with SCD1 being the predominant enzyme responsible for desaturation in all tissues, while SCD5 is primarily expressed in the pancreas and brain (Wang et al., 2005). The expression of SCD-1, is significantly upregulated and contributes to the progression of cancer. It catalyzes the conversion of saturated FA into ∆9-MUFA (AM et al., 2017). Noto et al. demonstrated that the gene encoding SCD1 exhibited the highest level of upregulation in lung tumor spheroidal cells with adherent cultures, and further revealed that SCD1 inhibitors selectively eradicated cells possessing stem-like properties (Noto et al., 2013). Additionally, their subsequent investigation demonstrated that SCD1 governs the regulation of stem cells in lung cancer by stabilizing and localizing transcriptional co-activators of the Hippo pathway effector yes-related proteins and PDZ-binding motifs (Noto et al., 2017a). The Hippo pathway, which is regulated by YAP and TAZ, has been shown to facilitate the renewal and differentiation of both embryonic and somatic stem cells (Cao et al., 2020). Meanwhile, another one of their studies confirmed that the expression of SCD1 was associated with a poor prognosis in lung adenocarcinoma, and inhibiting the activity of SCD1 reversed resistance to cisplatin in lung CSCs (Pisanu et al., 2017). The significance of MUFAs is further underscored by the heightened levels of SCD1 expression in lung, ovarian, breast, and GSCs, which aligns with the presence of MUFAs in CSCs (Li L. et al., 2013; Colacino et al., 2016; Lobello et al., 2016; Noto et al., 2017b). Furthermore, SCD1 also governs the Wnt signaling pathway in CSCs (El-Sahli et al., 2019) and has been observed to play a crucial role in the maintenance of stem cells in various other cancers, including melanoma, hepatocellular carcinoma (HCC), and colon cancer (Pisanu et al., 2018; Choi et al., 2019; Ma et al., 2019). However, it must be noted that certain cancer cells utilize an alternative pathway for FA desaturation and sapienate biosynthesis, bypassing the established SCD-dependent pathway and diminishing the relevance of SCD, thereby questioning its suitability as a therapeutic target (Vriens et al., 2019).

### 4.6 Transcriptional Induction-SREBPs

**FIGURE 4:**
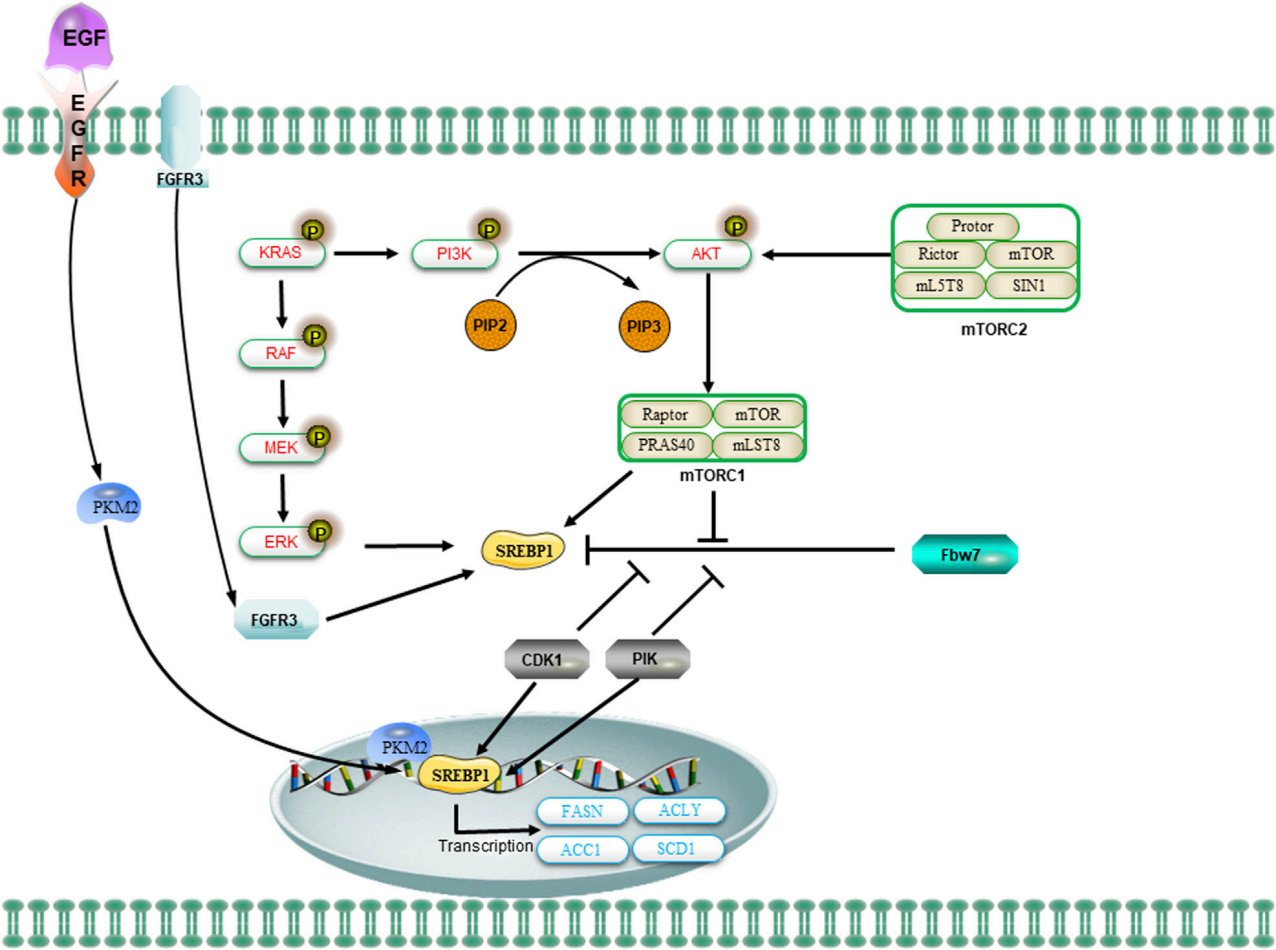
The regulation of SREBP1 and lipid metabolism in CSCs is mediated by oncogenic signaling. The oncogenic PI3K/Akt/mTORC1 and KRAS signaling pathways activate SREBP1 to facilitate *de novo* lipid synthesis and promote cellular growth. The mTOR signaling regulates the levels of SREBP1 through transcriptional or translational mechanisms. The activation of the PI3K/AKT/mTOR signaling pathway or FGFR3 results in the stabilization of SREBP1 proteins and facilitates their translocation to the nucleus. Additionally, the nuclear SREBP1 protein interacts with mitotic kinases Cdk1 and Plk1. The sequential phosphorylation of SREBP1 by Cdk1 and Plk1 impedes the interaction between the ubiquitin ligase Fbw7 and SREBP1, thereby attenuating the degradation of SREBP1. Upon activation of EGFR signaling, the nuclear form of PKM2 interacts with SREBP to facilitate the activation of SREBP target gene expression and promote lipid biosynthesis.

The synthesis and activation of FA in cancer cells can be accomplished through various mechanisms, including transcriptional induction. The transcriptional regulation of SREBPs governs the majority of enzymes involved in FA synthesis. The SREBPs are a group of transmembrane transcription factors that activate the expression of genes encoding enzymes essential for cholesterol synthesis and the production of UFA. Human cells contain three isoforms of SREBP, namely, SREBPla, SREBPic, and SREBP2. Among these isoforms, both SREBPla and SREBP1c are derived from individual genes through distinct transcription start sites (Shimano and Sato, 2017; DeBose-Boyd and Ye, 2018). The SREBP1 protein is a member of the SREBP family of transcription factors and serves as a key transcriptional regulator in adipogenesis, controlling the synthesis of FA and cholesterol (Li L. et al., 2013). SREBP1 is essential for maintaining lipogenesis in lipid and hypoxic conditions, and it directly regulates several key lipogenic enzymes, including ACLY, ACC1, and FASN (Pandey et al., 2013; Shimano and Sato, 2017) (Figure 4). Overexpression of SREBP1 has been observed in various human cancers, promoting the growth of a wide range of tumors and playing an essential role in maintaining the stemness of CSCs (Pandey et al., 2013). For instance, PR Pandey et al. demonstrated that the ectopic expression of SREBP1 in MCF10A cells significantly augmented stem cell adipogenesis and facilitated cellular proliferation and mammosphere formation (Pandey et al., 2013). The expression of oncogenic PI3K (H1047R) or K-Ras (G12V) in mammary epithelial cells induces *de novo* synthesis of adipose tissue, which necessitates the activation of sterol-regulatory element binding proteins (SREBP1 and SREBP2) within the PI3K/AKT/mTOR signaling pathway (Ricoult et al., 2016). In addition to promoting lipogenesis, SREBP1 also stimulates the expression of SCD1, thereby facilitating the production of MUFA (Lewis et al., 2015) (Figure 4). The silencing of SREBP1 results in the inhibition of proliferation and the induction of apoptosis in pancreatic cancer cells, thereby further suppressing lipid metabolism and impeding tumor growth *in vivo* (Sun et al., 2015). Furthermore, the growth of glioblastoma spheroids was significantly inhibited by blocking SREBP1 (Lewis et al., 2015). Mechanistically, during mitosis, SREBP1 protein hinders the interaction between the ubiquitin ligase Fbw7 and SREBP1, thereby suppressing the phosphorylation-mediated degradation of SREBP1 by Cdk1 and Plk1 (Bengoechea-Alonso and Ericsson, 2006; Bengoechea-Alonso and Ericsson, 2009; Bengoechea-Alonso and Ericsson, 2016). Furthermore, the PI3-kinase/Akt/rapamycin target (mTOR) C1 signaling pathway additionally promotes the nuclear accumulation of mature SREBP1 (Porstmann et al., 2008). Activation of EGFR signaling triggers the translocation of pyruvate kinase M2 (PKM2) into the nucleus, thereby inducing the Warburg effect (Christofk et al., 2008; Yang et al., 2011). Notably, nuclear PKM2 physically engages with SREBP1, contributing to enhanced lipid biosynthesis by stabilizing SREBP-1 proteins (Figure 4) (Zhao X. et al., 2018). These findings provide further evidence for the interplay between glycolysis and FA metabolism.

## 5 Characterization of FA metabolism in CSCs

Recently, the significance of lipid metabolism in cancer cells has been consistently emphasized, leading to a series of notable advancements that offer valuable reference indices and guidance for cancer therapy (Luo X. et al., 2017). The energy metabolism of CSCs is mainly carried out in mitochondria, and they are able to efficiently utilize nutrients, such as FA, to produce ATP to provide the energy they need in the harsh microenvironment. The high level of UFA in CSCs has been demonstrated, and it has been shown that inhibiting the activities of SCD1 and acetaldehyde dehydrogenase 1A1 (ALDH1A1), or reducing the level of NF-κB in CSCs, can significantly decrease UFA content, diminish the stemness of CSCs, and impede tumor formation (Sun and Yang, 2019). The level of unsaturated fatty acid (UFA) in ovarian CSCs is significantly elevated compared to non-CSCs, and key lipases involved in FA synthesis, including ACLY, ACC, and FASN, exhibited elevated levels (Li et al., 2017). These lipases were regulated by the lipid-generating transcription factor SREBP1c, which has gradually emerged as a reliable marker of stemness in CSCs (Visweswaran et al., 2020). Additionally, the activity of lipase activity was modulated by other protein kinases, such as the reduction in AMP kinase (AMPK) phosphorylation, and content in CSCs not only elevated lipase activity but also increased malonyl coenzyme A levels, a precursor for fatty acid synthesis, resulting in enhanced fatty acid synthesis and mitochondrial β-oxidation (Bort et al., 2020). Furthermore, increased FA synthesis facilitates the uptake and utilization of lipids by CSCs (Peck and Schulze, 2016). Conversely, inhibition of the NF-κB-regulated lipid desaturase signaling pathway can effectively eradicate CSCs and impede their tumorigenic potential (Li et al., 2017). Additionally, the dynamic equilibrium of FA is pivotal for lipid metabolism in CSCs, and maintaining a stable metabolic state in CSCs contributes to chemoresistance and the acquisition of stem cell-like properties (Wang et al., 2018).

The connection between glycolipid metabolism in CSCs lies in the fact that AcCoA, generated through the oxidation of pyruvate—an intermediate product of glycolipid metabolism—can be utilized for FA synthesis, thereby facilitating the self-renewal of CSCs and contributing to the maintenance of their stemness (Pizer et al., 2000). Studies have demonstrated that maintaining a well-balanced ratio between FA and glycerophospholipids can effectively impede the progression of HCC (Lin et al., 2017). The elevated glucose metabolism and β-oxidation in CSCs of HCC and leukemia ensure the provision of alternative energy sources under extreme conditions, thereby sustaining their stemness (Chen C. L. et al., 2016). The intermediates generated through glycolysis can also serve as substrates for the synthesis of FA, thereby facilitating the self-renewal of CSCs (Corominas-Faja et al., 2014). Thus, the maintenance of pluripotency, self-renewal, proliferation, and formation of CSCs relies on the delicate balance of FA homeostasis or the state of catabolism/anabolism.

## 6 Characterization of cholesterol metabolism in CSCs

**FIGURE 5:**
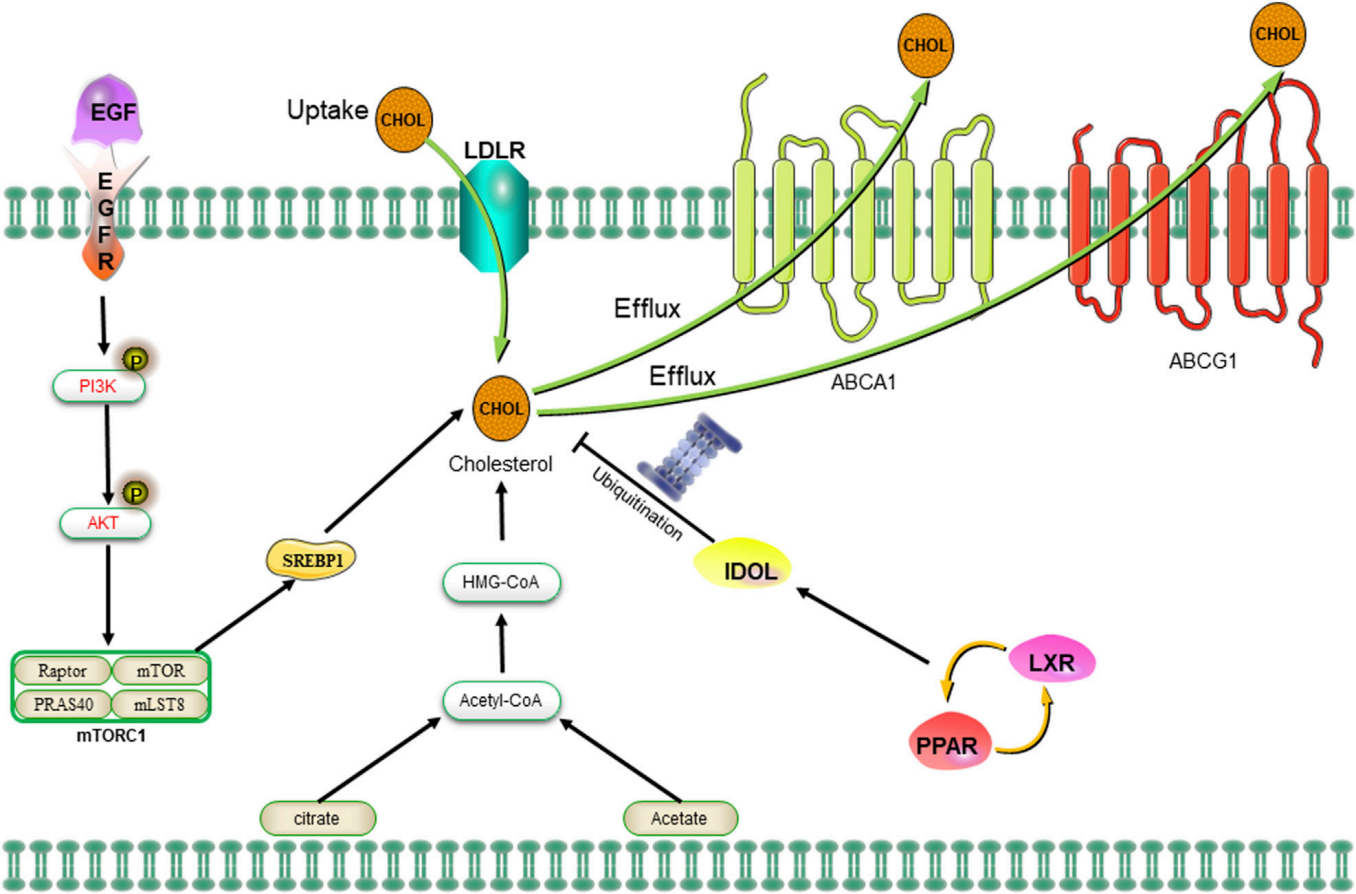
The uptake of cholesterol is facilitated by LDLR via the epidermal growth factor receptor-dependent pathway. The MVA pathway is responsible for cholesterol synthesis, while LDLR plays a crucial role in both the negative regulation of cholesterol uptake and the control of cholesterol efflux. Abbreviation: EGFR, epidermal growth factor receptor; LDLR, low density lipoprotein receptors; ABCA1, ATP-binding cassette transporter 1; PI3K/Akt, phosphatidylinositol 3-kinase/protein kinase B; SREBP-1, sterol regulatory element binding proteins; MVA, mevalonate; IDOL, inducible degrader of the low-density lipoprotein receptor; PPAR/LXR, lipid-activated transcription factors/LXRs.

The sources of cholesterol can be categorized as either exogenous or endogenous, with exogenous cholesterol originating from dietary intake and endogenous cholesterol being synthesized within the body. The maintenance of cholesterol homeostasis is dependent on two primary mechanisms (Hafiane et al., 2019). On one hand, cholesterol levels can be elevated through the re-synthesis of AcCoA provided by glycolysis, glutamine metabolism, the TCA cycle, or exogenous uptake of low-density lipoprotein (LDL) receptors. Additionally, peripheral cholesterol can return to the liver in the form of LDL via the cholesterol reverse transporter, as well (Munir et al., 2019). On the contrary, cholesterol levels can be downregulated through inhibition of the MVA pathway or activation of liver X receptors (LXRs). The MVA pathway can be attenuated by protein hydrolysis or nuclear importation of SREBP2, while LXRs can be stimulated by the conversion of cholesterol to oxysterols (Ahmad et al., 2019). The activation of the LXRs/PPAR pathway subsequently induces the transcription of the E3 ubiquitin ligase IDOL, which in turn facilitates the ubiquitination of LDLR and enhances the expression of cholesterol efflux pumps ABCA1 and ABCG1 (Figure 5) (Spite, 2014). The MVA pathway plays a crucial role in the biosynthesis of steroid hormones, cholesterol, and nonsteroidal isoprenoids. The pathway primarily maintains homeostasis in the microenvironment of CSCs through protein geranylgeranylation. Additionally, 3-hydroxy-3-methylglutaryl monoacyl-coenzyme A (HMG-CoA) serves as the rate-limiting enzyme in the MAV pathway and represents a molecular target for statin drugs. The HMG-CoA reductase, which is the rate-limiting enzyme in the MAV pathway, serves as a molecular target for statin-type cholesterol-lowering drugs. The administration of statin drugs disrupts the MAV pathway, thereby inhibiting geranylgeranylation of proteins and disrupting the homeostasis of the microenvironment in CSCs, ultimately leading to their eradication (Mullen et al., 2016). For example, an overexpression of HMG-CoA is revealed in basal-like tumors, and the inhibition of the MAV pathway through simvastatin demonstrated a significant reduction in the number of CSCs within the tumors (Mancini et al., 2018). The combination of valproic acid and simvastatin can concurrently modulate the MAV pathway and AMPK phosphorylation level, thereby inhibiting the YAP oncogene. This enhances the sensitivity of denervation-tolerant prostate cancer cells to doxorubicin while reducing drug resistance caused by CSCs (Iannelli et al., 2020). Furthermore, the treatment of colorectal cancer cell lines with metformin, an AMPK activator, an HMG-CoA reductase inhibitor, or a mammalian target of rapamycin (mTOR) inhibitor significantly decreased the population of CSCs; however, the number of CSCs rebounded after treatment with mevalonic acid, indicating that mevalonic acid attenuates the inhibitory effect of these treatments on CSCs (Sharon et al., 2015). Moreover, bile acids and oxysterols serve as two chemical by-products of the MVA pathway. They function as ligands for a variety of nuclear receptors (including FXR, VDR, LXR, and PXR) as well as G-protein coupled receptors (Joyce and Gahan, 2016; Hafiane et al., 2019). Ting Fu et al. discovered that in colorectal cancer, a high-fat diet and dysregulated WNT signaling pathway led to alterations in bile acid profiles, activation of FXR, and the initiation of malignant transformation in Lgr5+ subpopulation CSCs (Fu et al., 2019). Similarly, the MAV pathway is found to enhance the proliferation of pancreatic CSCs, while the administration of atorvastatin effectively inhibits this proliferative effect (Brandi et al., 2017). Notably, in the MAV pathway, the inhibition of geranylgeranyltransferase can effectively reduce CSCs by suppressing protein isoprenylation. However, it has been observed that inhibiting cholesterol synthesis with squalene synthase inhibitors does not lead to a decrease in the number of CSCs (Brandi et al., 2017). The formation of breast tumorspheres occurs during the culture of breast CSCs, giving rise to both breast cancer cells and breast CSCs (Gupta et al., 2009). The inhibition of cholesterol synthesis effectively reduces the formation of breast tumorspheres, indicating that targeting the cholesterol synthesis pathway is a promising therapeutic strategy for suppressing the development of breast cancer by specifically targeting CSCs (Ehmsen et al., 2019). Some researchers have also demonstrated that synthetic progestins, such as medroxyprogesterone acetate (MPA), widely utilized in clinical practice, expedite the formation of breast tumorspheres while simultaneously increasing the activity of ALDH1A1 in CSCs and augmenting the tumorigenicity of CSCs (Goyette et al., 2017). Furthermore, it has been found that cholesterol synthesis inhibitors can mitigate this undesirable induction of breast tumorspheres by MPA (Liang et al., 2017).

## 7 The impact of autophagy on lipid metabolism in CSCs

Autophagy is a natural and highly conserved cellular degradation process that involves the lysosome-mediated breakdown of unwanted or dysfunctional intracellular components, including molecules and organelles (Mahapatra et al., 2021). The process of autophagy is crucial for maintaining cellular homeostasis, and any disruption to this mechanism may potentially facilitate the development of tumorigenesis. The process of lipophagy involves the fusion of LDs phagosomes with lysosomes to form autophagolysosomes, followed by the breakdown of LDs to generate free FA (Zhang et al., 2022). These FAs are then transported to the mitochondria for β-oxidation, resulting in energy production (Singh et al., 2009). The process of autophagy serves as a metabolic adaptation employed by tumor cells to surmount nutrient deprivation (Sinha et al., 2017). Additionally, autophagy facilitates the mobilization of nutrients and confers survival advantages to cancer cells, particularly under conditions of cellular stress such as hypoxia, chemotherapy, and radiotherapy (Lin et al., 2023). It plays a crucial role in maintaining cellular homeostasis and enables cells to withstand disturbances within the TME (Camuzard et al., 2020). The process of autophagy is crucial for maintaining lipid homeostasis, and the inhibition of autophagy leads to a decrease in the rate of β-oxidation in LDs, thereby reducing energy utilization (Li et al., 2018). Furthermore, autophagy facilitates the survival of CSCs by providing energy, enabling them to swiftly adapt to the challenging microenvironment post-chemotherapy, such as CSCs exhibit a higher autophagic rate compared to normal stem cells, and inhibition of autophagy compromises the stemness and tumorigenicity of CSCs (Visweswaran et al., 2020). The induction of autophagy is governed by the UNC-51-like autophagy-activated kinase 1 (ULK1) complex, and both mTOR and AMPK have the ability to modulate the expression level of ULK1. The activation of AMPK and the inhibition of mTOR can effectively suppress cholesterol synthesis and downregulate ULK1, thereby inducing apoptosis in CSCs (Bu et al., 2020). Moreover, the basal level of autophagy/mitophagy is higher in BCSCs compared to normal tissue-specific stem cells and autophagy induces the upregulation of CD44 and vimentin, both of which are recognized as stem cell markers (Cufi et al., 2011). GSCs also exhibit high expression levels of the autophagy regulators SQSTM1 and DRAM1, which are positively correlated with the expression of mesenchymal factors such as c-MET (Galavotti et al., 2013). The involvement of CD133, another stem cell marker, in the regulation of autophagy in GSC has been observed, as evidenced by the fact that the cytoplasmic localization of CD133 is relatively enhanced under conditions of glucose deprivation, while it remains membrane-bound in the presence of normal glucose levels within the cells (Sun H. et al., 2016). Autophagy involves several specific membrane structures, including phagosomes, autophagolysosomes, and autolysosomes. These structures are capable of engulfing isolated cytoplasmic constituents and subsequently degrading intracellular substances through hydrolytic enzymes (Klionsky et al., 2014). The lipid metabolism alterations in CSCs facilitate the regeneration of these membrane structures, and reciprocally, the regenerated membrane structures exert an influence on the CSCs, thereby establishing a positive feedback loop (Snaebjornsson et al., 2020). Moreover, the maintenance of pluripotency in breast hematopoietic stem cells under various pathophysiological conditions is also attributed to the crucial role played by autophagic homeostasis (Han et al., 2018). Interestingly, in addition to providing energy to CSCs, autophagy also facilitates the lipid peroxidation of UFA, which is abundantly expressed on the cellular membrane, leading to programmed cell death - ferroptosis (Xie et al., 2020). Ours and other studies have indicated that facilitating ferroptosis can specifically kill CSCs (Mai et al., 2017; Polewski et al., 2017; Ni et al., 2021; Yang Y. et al., 2022). However, it must be admitted that the potential benefits and drawbacks of autophagy in promoting the survival of CSCs necessitate further investigation: On the one hand, autophagy may facilitate CSC survival during periods of nutrient deprivation by recycling cellular components, providing an alternative energy source for their maintenance; On the other hand, excessive or dysregulated autophagy could lead to the degradation of essential cellular components in CSCs, compromising their functionality and survival.

## 8 The involvement of CSCs in crucial signaling pathways of lipid metabolism

The maintenance of stemness, survival, proliferation, and invasion in CSCs involves a variety of lipid metabolic pathways, including the Notch, Hippo, Hh, and Wnt signaling pathways (Wang et al., 2022). The pathways through which stem cells can be derived via genetic mutations and epigenetic alterations have a significant potential to be exploited for the maintenance of unrestricted proliferation, invasion, and drug resistance (Reya et al., 2001; Wang et al., 2022).

### 8.1 Wnt signaling pathway

The Wnt signaling cascade comprises three primary pathways: the canonical Wnt pathway (which results in β-catenin accumulation, activation of transcription-activation complexes, and involvement in tumorigenesis), the non-canonical planar cell polarity pathway, and the non-canonical Wnt-calcium pathway (Takebe et al., 2015). In the typical Wnt pathway, Wnt ligands bind to the frizzled family of transmembrane receptors, thereby activating disheveled. This activation then collaborates with the T-cell factor/LEF family to induce stabilization and accumulation of nuclear β-catenin transcriptional activity. The Wnt signaling pathway exhibits a high degree of evolutionary conservation during the development of embryonic proliferative tissues, such as the hematopoietic system, skin, and intestines. This conservation is evident in terms of somatotaxis stereotypy, cell fate specification, cell proliferation, and migration (Teo et al., 2006). During tumorigenesis, the Wnt signaling facilitates tumor migration and invasion by upregulating genes involved in cell adhesion, such as Eph/ephrin, E-cadherin, and MMPs (Lang et al., 2019). Thus, the Wnt signaling pathway plays a pivotal role in the regulation of CSCs (Patel et al., 2019; Fendler et al., 2020). The Wnt signaling pathway, for instance, is also implicated in lipid synthesis within CSCs. Specifically, the canonical Wnt/β-catenin pathway governs subordinate lipogenesis and MUFA production (Bagchi et al., 2020). Additionally, the Wnt/β-catenin signaling significantly regulates the process of *de novo* adipogenesis in breast cancer cells, characterized by a substantial upregulation of ACC, FASN, and SREBP1-c expression (Vergara et al., 2017). Another study suggests that SCD potentially links Wnt signaling and lipid metabolism as in mouse liver hematopoietic stem cells, the Wnt/β-catenin pathway regulates SCD expression, and SCD-derived monounsaturated fatty acids create a positive feedback loop, reinforcing Wnt signaling through enhanced Lrp5/6 mRNA stability and expression (Lai et al., 2017). Furthermore, MUFAs play a crucial role in the synthesis and release of Wnt ligands (Rios-Esteves and Resh, 2013). The metabolism of FA in YAP/TAZ signaling, specifically the function of SCD1, is reliant on the activity of the β-catenin pathway in CSCs (Noto et al., 2017a). The dysregulation of the Wnt signaling pathway and a high-fat diet in colorectal cancer lead to alterations in bile acid distribution, activation of FXR, and subsequent malignant transformation of the Lgr5+ subpopulation of CSCs. This process promotes the progression from adenomas to adenocarcinomas (Fu et al., 2019).

### 8.2 Notch signaling pathway

The Notch signaling pathway comprises of Notch receptors, DSL proteins (Notch ligands), CSLs (CBF-1, Hairless Suppressor Factor, Lag), DNA-binding proteins, other effectors, and Notch-regulated molecules. The Notch signaling pathway is a highly conserved signal transduction pathway that plays a crucial role in various biological processes, including tumor metastasis and immune evasion (Takebe et al., 2015; Bocci et al., 2019). The activation of the Notch pathway in CSCs has been demonstrated by numerous studies to promote cell survival, self-renewal, and metastasis while inhibiting apoptosis. The aberrant activation of Notch signaling (Notch1 and Notch4) facilitates the self-renewal and metastasis processes in breast cancer and HCC stem cells (Stylianou et al., 2006; Harrison et al., 2010). In lipid metabolism, the Notch signaling pathway regulates the expression of peroxisome proliferator-activated receptor α and lipid oxidation genes to maintain lipid homeostasis and redox homeostasis in lipid metabolism (Song et al., 2016). The selective elimination of colon CSCs through inhibition of Notch signaling is achieved by targeting scd1-dependent lipid desaturation (Yu et al., 2021). Furthermore, in *Drosophila*, the Notch signaling pathway is responsive to environmental sterol levels and its expression is regulated by dietary cholesterol, ultimately leading to the differentiation of enterocytes from a stem-like state (Obniski et al., 2018). The activation of the Notch signaling is also regulated by dietary cholesterol, thereby inducing the differentiation of enterocytes from a stem-like state. Additionally, the Notch pathway plays a crucial role in angiogenesis, EMT, and the proliferation of CSCs within cancer cells. It has been observed that a low-sterol diet can potentially restrict the growth of enteroendocrine tumors by attenuating the Notch response (Obniski et al., 2018). Kalucka et al., 2018 discovered that Notch1 regulates FAO to maintain intermediate lipid homeostasis and redox homeostasis in CSCs. Importantly, the Notch signaling in humans is influenced by the lipid composition of the cellular membrane (Sorrentino et al., 2014).

### 8.3 Hippo signaling

The evolutionarily conserved Hippo signaling pathway is initiated by Hippo kinase, a serine/threonine kinase. In normal conditions, Hippo kinase is inactive due to phosphorylation. However, external stimuli activate Hippo kinase, leading to the phosphorylation and activation of downstream molecules MST1/2. Activated MST1/2, in turn, phosphorylate LATS1/2, exerting inhibitory effects on the activities of YAP and transcriptional coactivator with PDZ-binding motif (Mo et al., 2014). The activation of YAP or TAZ in CSCs has been confirmed to sustain the self-renewal and tumor-initiating capacity (Bhat et al., 2011), promote cellular pluripotency (Cordenonsi et al., 2011) and drug resistance (Huo et al., 2013), and exhibits a strong correlation with the process of EMT (Kulkarni et al., 2018; Sundqvist et al., 2018). Emerging evidence suggests a close and significant association between the Hippo-YAP/TAZ signaling and the regulation of lipid metabolism in cancer stemness (Shu et al., 2019). For example, SCD1, a major regulator of MUFA, contributes to the maintenance of cancer stemness by modulating the expression and nuclear localization of YAP/TAZ (Noto et al., 2017a). GGPP, an intermediate in the control of the MAV pathway, stabilizes YAP/TAZ (Sorrentino et al., 2014). As previously mentioned, SCD1 plays a crucial role in regulating the stemness of lung CSCs by stabilizing YAP/TAZ and facilitating their nuclear localization (Noto et al., 2017a). Notably, a positive feedback loops involving LATS2 and p53 has been identified to inhibit cholesterol synthesis (Aylon et al., 2016). Additionally, LATS2 binds to endoplasmic reticulum tethered precursors (P-SREBP) of SREBP1 and SREBP2, thereby suppressing the transcription of SREBP mRNAs and subsequently inhibiting cellular SREBP activity (Aylon and Oren, 2016). Recent studies have demonstrated that the pro-carcinogenic properties of YAP/TAZ are contingent upon cholesterol biosynthesis activity and MVA-dependent nuclear localization and activity of YAP/TAZ (Sorrentino et al., 2014). The lipid synthesis mediated by YAP/TAZ may serve as a crucial factor influencing metabolic alterations in CSCs (Koo and Guan, 2018).

### 8.4 Hh signaling

The Hh signaling network is intricate and encompasses extracellular Hh ligands, the transmembrane protein receptor PTCH, the transmembrane protein SMO, intermediate signal transduction molecules, and the downstream effector molecule GLI (Yang et al., 2020). The membrane protein SMO exerts a positive regulatory function, while the transmembrane protein PTCH plays a negative regulatory role. The GLI protein acts as an effector, and in vertebrates, it exists in three isoforms: Gli1, Gli2, and Gli3. These effector proteins exhibit distinct functionalities: Gli1 exerts a robust transcriptional activation effect, whereas Gli3 functions as a transcriptional inhibitor. On the other hand, Gli2 displays dual functionality by both activating and inhibiting transcription but primarily serves as a transcriptional activator (Sasaki et al., 1999; Li S. H. et al., 2013). The Hh signaling pathway also plays a pivotal role in the regulation of CSCs. It governs the proliferation of postnatal mammary stem cells and orchestrates the intricate ductal architecture formation in the adult mammary gland (Miyazaki et al., 2016). The existing evidence suggests that lipid metabolism also plays a regulatory role in the Hh signaling and its ligand properties, highlighting the crucial involvement of lipids as key regulators in Hh signaling (Blassberg and Jacob, 2017). The covalent modification of SMO by cholesterol is regulated by the Hh signaling pathway and plays a crucial role in mediating Hh signaling and cellular biological functions (Radhakrishnan et al., 2020). PTCH1 inhibits the cholesterol modification of SMO, while SHH overexpression enhances it (Hu and Song, 2019). Furthermore, SMO exerts direct or indirect inhibition on FA and cholesterol synthesis by activating adenosine monophosphate kinase through a non-classical pathway (Blassberg and Jacob, 2017). Notably, recent clinical trials have utilized SMO and GLI inhibitors to target the Hh signaling pathway (Long et al., 2016; Zhang et al., 2016). Thus, the Hh signaling pathway, intricately linked with lipid metabolism and CSCs, demonstrates the regulatory role of lipids in signaling cascades, offering potential therapeutic targets for cancer intervention.

## 9 Emerging drugs that target lipid metabolism in CSCs

The targeted eradication of CSCs can be accomplished by intervening in various aspects of their lipid metabolism, including lipogenesis, lipid uptake, lipid desaturation, and FA oxidation (Yi et al., 2018). Due to the exorbitant cost and inherent risks associated with the discovery and development of novel therapeutic agents, there has been a growing interest in drug repositioning strategies for hard-to-treat diseases. This approach offers an opportunity to establish effective targeting strategies aimed at eradicating CSCs. The targeted elimination of CSCs can be achieved by disrupting various aspects of lipid synthesis, including FA synthesis, lipid desaturation, and cholesterol synthesis (Table 2).

**TABLE 2:** Emerging drugs that target lipid metabolism in CSCs.

Metabolism type	Drug	Targeting enzyme	CSC or tumor type	Metabolic processes or signaling pathways	Clinical trial
Lipogenesis	Resveratrol	FASN	BCSCs (Pandey et al., 2011), GCSCs (Sayd et al., 2014), PCSCs (Subramaniam et al., 2018)	Regulation of FASN	Clinical Trial
	Orlistat	FASN	NSCLC (Ali et al., 2018)	Regulation of FASN	failure
	TVB-2640	FASN	NSCLC and breast cancer (Corominas-Faja et al., 2014)	Regulation of FASN, Inhibitor of HMG-COAR, inhibitor of cholesterol synthesis	Recruiting
	Cerulenin	FASN	PCSCs (Brandi et al., 2017), GCSCs (Yasumoto et al., 2016)	Regulation of FASN	Pre-clinical
	Leptin	ACC	BCSCs (Schug et al., 2015)	TAK1-AMPK signaling pathways	Pre-clinical
	Soraphen A	ACC	BCSCs (Corominas-Faja et al., 2014)	Regulation of FASN	Pre-clinical
	ND-646	ACC	Non-small-cell lung CSCs (Svensson et al., 2016)	Regulation of FASN	Pre-clinical
FAO	ST1326	CPT1A	Lymphoma (Pacilli et al., 2013), AML cells (Kalucka et al., 2018)	Inhibition of FAO	Pre-clinical
	Avocatin B	FABP4	AML cells (Tabe et al., 2018)	enhanced glucose uptake	Pre-clinical
	Etomoxir	CPT1A	TNBC (Camarda et al., 2016), leukemia (Samudio et al., 2010)	Inhibition of FAO	Pre-clinical
Cholesterol synthesis	25-HC or fatostatin, Pyrvinium pamoate	SREBPs	Colon CSCs (Wen et al., 2018), TNBC CSCs (Dattilo et al., 2020)	FA synthesis and cholesterol synthesis	Pre-clinical
	Simvastatin	HMGCR	BCSCs (Dattilo et al., 2020)	Cholesterol synthesis	FDA-approved cardiovascular system drug
Lipid desaturation	MF-438	SCD1	Colon CSCs (Yu et al., 2021), lung CSCs (Pisanu et al., 2017)	Regulation of Wnt, Notch, and YAP/TAZ signaling pathways	Pre-clinical
	A939572	SCD1	CRC (Chen et al., 2016c), clear cell renal cell carcinoma (von Roemeling et al., 2013), Liver cancer (Tracz-Gaszewska and Dobrzyn, 2019)etc.	MUFA synthesis	Pre-clinical
	SSI-4	SCD1	Liver CSCs (Belkaid et al., 2015)	The inhibition of SCD1 compels hepatic CSCs to undergo differentiation by inducing ER stress	Pre-clinical
	CAY10566	SCD1	Ovarian CSCs (Li et al., 2017), glioblastoma CSCs (Valle et al., 2020)	NF-κB pathway, ER stress	Pre-clinical
	PluriSIn#1	SCD1	Colon CSCs (Yuan et al., 2022), liver CSCs (Lobello et al., 2016)	Wnt/β-catenin and Notch signaling	Pre-clinical
	T-3764518	SCD1	CRC (Nishizawa et al., 2017)	Inhibit lipogenesis	Pre-clinical
	SC-26196	Delta 6 desaturase	Ovarian CSCs (Li et al., 2017)	Inhibit polyunsaturated FA synthesis	Pre-clinical
	BetA	SCD1	CRC-CSCs (Potze et al., 2016)	BetA inhibits SCD1-induced rapid death of colonic hematopoietic stem cells	Pre-clinical
Lipid uptake	CD36 Antibody	CD36	OSCC (Pascual et al., 2017)	Inhibit lipogenesis	Pre-clinical

BCSCs: breast cancer CSCs, GCSCs: glioblastoma CSCs, PCSCs: pancreatic CSCs, FASN: FA synthase, NSCLC: non-small cell lung cancer, AML: acute myeloid leukemia, TNBC: triple-negative breast cancer, ER: endoplasmic reticulum, CRC colorectal cancer, OSCC oral squamous cell carcinomas.

The FASN gene is the primary focus among adipogenic genes and the antitumor activity of several FASN inhibitors has been demonstrated in preclinical cancer models. The expression of invasiveness, sphere formation, and stemness markers is effectively reduced by both FASN inhibitors and RNA silencing, leading to the eradication of various CSCs (Table 2) (Yasumoto et al., 2016; Brandi et al., 2017). The development of novel FASN inhibitors is underway, and preliminary clinical trial data on TVB-2640 demonstrate its potential to mitigate tumor response in patients with non-small cell lung cancer and breast cancer when used in combination with paclitaxel (Jones and Infante, 2015). Similarly, the formation of mammospheres was inhibited by Soraphen A, an inhibitor of ACC (Corominas-Faja et al., 2014). Mechanistically, Soraphen A inhibits the self-renewal and growth of CSC-like cells by blocking FA synthesis and abolishes the promotion of CSC proliferation mediated by human epidermal growth factor receptor 2 (HER2) (Corominas-Faja et al., 2014). The inhibition of ACC not only suppressed tumor growth, metastasis, and recurrence in non-small cell lung and breast cancers, but also underscored the significance and potential of ACC in suppressing CSCs and combating cancer (Schug et al., 2015; Svensson et al., 2016).

Additionally, the selective elimination of CSCs can be achieved by targeting SCD1, an enzyme responsible for the conversion of fully saturated FA into MUFAs. The recent demonstration of SCD in a genetic mouse model is particularly noteworthy, as it is essential for the emergence of tumor-initiating cells (Lai et al., 2017). The significant reliance on UFA renders SCD a promising target for the eradication of CSCs (Peck and Schulze, 2016). The SCD1 inhibitors, such as CAY10566 and A939572, have been documented to effectively suppress cancer stemness, inhibit tumorigenesis, and overcome chemoresistance in cancer cells (Tracz-Gaszewska and Dobrzyn, 2019). Notably, MF-438 and PluriSIn #1, as inhibitors of SCD1, exhibited selective eradication of colonic CSCs while showing no efficacy against cancer cells (Yu et al., 2021). Furthermore, the inhibition of SCD1 expression enhances the sensitivity of CSCs to cisplatin and reduces the occurrence of drug resistance (Pisanu et al., 2017). The SSI-4 compound is a novel inhibitor of SCD1 that effectively overcomes sorafenib resistance in hepatic CSCs. The antitumor activity of SSI-4 against hepatic CSCs was also observed in animal models, with no significant adverse effects reported (Ma et al., 2017). Importantly, the combination of SSI-4 and sorafenib demonstrated the most significant inhibition of tumorigenesis in the sorafenib-resistant PDTX model. The combination of SCD1 inhibitors and chemotherapy may potentially offer a more efficacious therapeutic approach. The effective SCD1 inhibitors utilized in the preclinical phase are summarized in Table 1.

The reliance of CSCs on FAO justifies the targeting of these cells with FAO inhibitors. The compound Etomoxir functions as a highly specific inhibitor of mitochondrial CPT1A (Holubarsch et al., 2007) (as shown in Table 1). The inhibition of FAO by etomoxir in leukemic CSCs leads to the suppression of cell proliferation and enhances the susceptibility of human leukemic cells to apoptosis (Samudio et al., 2010; Estan et al., 2014). ST1326 is a CPT1A inhibitor that suppresses FAO and exhibits cytotoxic activity against leukemia cell lines, while sparing normal CD34^+^ myeloid cells (Ricciardi et al., 2015) (Table 1). ST1326 also exhibits preventive effects against MYC-driven lymphomagenesis in an Eμ-myc transgenic mouse model (Pacilli et al., 2013).

The activation of cholesterol synthesis has been previously suggested to be linked with the invasive and metastatic capabilities of CSCs. The inhibition of SREBP activation by 25-HC or adiponectin hampers adipogenesis, including FA and cholesterol synthesis, and disrupts the expression of genes associated with CSCs (Sun H. W. et al., 2016). Furthermore, Dattilo R not only significantly inhibits lipid anabolism in CSCs but also exhibits a cytotoxic effect on TNBC -CSCs by suppressing cholesterol synthesis in TNBC (Dattilo et al., 2020). The inhibition of cholesterol biosynthesis by Simvastatin significantly suppresses the formation and growth of mammospheres (Gu et al., 2019). Additionally, statins exert their effects on CSCs by inhibiting signaling pathways associated with protein farnesylation and protein geranylation in the MVA pathway (Ginestier et al., 2012; Iannelli et al., 2020). Similarly, metformin suppresses colorectal CSCs by inhibiting protein prenylation of the MVA pathway (Seo et al., 2020). Taken together, these emerging drugs targeting lipid metabolism in CSCs offer promising avenues for selective elimination of CSCs, focusing on disrupting various aspects such as lipogenesis, lipid uptake, desaturation, and fatty acid oxidation, and presenting potential strategies to suppress CSC proliferation and enhance therapeutic outcomes.

## 10 Conclusion

In conclusion, the intricate interplay between lipid metabolism dynamics and CSCs has emerged as a pivotal determinant in the initiation, progression, and therapeutic response of various cancers. The preservation of CSC stemness relies heavily on unique alterations in lipid metabolism, encompassing strategies to increase intracellular fatty acid content, promote β-oxidation for optimized energy utilization, and upregulate the MAV pathway for efficient cholesterol synthesis. Additionally, the versatile role of LDs as alternative energy sources in the context of glycolysis blockade underscores their significance in safeguarding FAs from peroxidation. The symbiotic relationship between autophagy and lipid metabolism not only facilitates the rapid adaptation of CSCs to the harsh microenvironment induced by chemotherapy but also presents a complex network of potential therapeutic targets. As we delve into the molecular intricacies of lipid metabolism in CSCs, a myriad of possibilities emerges for innovative cancer therapies. Identifying and targeting key components within this dynamic metabolic landscape holds promise for disrupting CSC functions, curbing tumor initiation, and overcoming drug resistance.

In recent years, the rapid advancement of high-throughput technologies has significantly enhanced our comprehension of the role of metabolic reprogramming in oncogenesis, which is now widely acknowledged as a fundamental characteristic of cancer. CSCs play a pivotal role in the initiation, progression, distant dissemination, and acquisition of drug resistance in tumors. Metabolic alterations have been shown to serve as the primary mechanism through which cancer cells and CSCs evade adverse environmental influences. In lipid metabolism, such as augmented FA uptake, neoadipogenesis, LDL formation, FAO, and lipid desaturation, play a significant role in the generation and sustained maintenance of CSCs. Moreover, lipid synthesis plays a crucial role in the activation of several pivotal oncogenic signaling pathways, including the Notch, Wnt/β-catenin, Hippo, and HH signaling. The targeting of crucial lipid synthesis molecules holds great potential in the elimination of CSCs. In addition, the heterogeneity and metabolic plasticity of CSCs pose a dilemma, despite the promising opportunity to target cellular metabolism for their elimination (Wang et al., 2013; Thomas and Majeti, 2016; Krstic et al., 2017). The metabolic profiles of CSCs and tumor cells can be modulated in response to nutrient availability. For instance, when cetuximab counteracted the Warburg effect, HNSCC cells exhibited elevated levels of ACC, thereby reprogramming cancer metabolism from glycolysis to adipogenesis in order to support energy demands and facilitate proliferation (Luo J. et al., 2017). The enhanced uptake of FA, neoadipogenesis, LDL formation, FAO, and lipid desaturation has been extensively documented in CSCs across various cancer types. The majority of the targeted compounds, however, have not demonstrated therapeutic efficacy in preclinical cancer models, with only a limited number of inhibitors progressing to clinical trials. The metabolic flexibility of CSCs poses a challenge in effectively eliminating these cells through a single metabolic pathway, necessitating the exploration of synergistic targeting of multiple metabolic pathways in future studies.

In conclusion, the intricate interplay between lipid metabolism dynamics and CSCs has emerged as a critical determinant in various cancers’ initiation, progression, and therapeutic response. The preservation of CSC stemness heavily relies on unique alterations in lipid metabolism, including strategies to increase intracellular fatty acid content, promote β-oxidation for optimized energy utilization, and upregulate the mevalonate pathway for efficient cholesterol synthesis. Additionally, the versatile role of LDs as alternative energy sources in the context of glycolysis blockade underscores their significance in safeguarding fatty acids from peroxidation. The symbiotic relationship between autophagy and lipid metabolism not only facilitates the rapid adaptation of CSCs to the harsh microenvironment induced by chemotherapy but also presents a complex network of potential therapeutic targets. As we delve into the molecular intricacies of lipid metabolism in CSCs, a myriad of possibilities emerges for innovative cancer therapies. Identifying and targeting key components within this dynamic metabolic landscape holds promise for disrupting CSC functions, curbing tumor initiation, and overcoming drug resistance.

All in all, this comprehensive review underscores the potential of lipid metabolism as a therapeutic nexus, offering valuable insights into the vulnerabilities of CSCs. As we move forward, unraveling the complexities of lipid-mediated stemness maintenance opens avenues for the development of targeted interventions, marking a significant stride in the pursuit of effective cancer treatments.
